# Seoul Virus Associated with Pet Rats, Scotland, UK, 2019

**DOI:** 10.3201/eid2710.211298

**Published:** 2021-10

**Authors:** James G. Shepherd, Andrew E. Blunsum, Stephen Carmichael, Katherine Smollett, Hector Maxwell-Scott, Eoghan C.W. Farmer, Jane Osborne, Alasdair MacLean, Shirin Ashraf, Rajiv Shah, Rory Gunson, Ana da Silva Filipe, Emma J. Aarons, Emma C. Thomson

**Affiliations:** MRC–University of Glasgow Centre for Virus Research, Glasgow, Scotland, UK (J.G. Shepherd, S. Carmichael, K. Smollett, S. Ashraf, R. Shah, A. da Silva Filipe, E.C. Thomson);; National Health Service Greater Glasgow and Clyde, Glasgow (A.E. Blunsum, E.C.W. Farmer);; Public Health England, London, UK (H. Maxwell-Scott, J. Osborne, E.J. Aarons);; West of Scotland Specialist Virology Centre, Glasgow (A. MacLean, R. Gunson);; London School of Hygiene and Tropical Medicine, London (E.C. Thomson)

**Keywords:** hantavirus infections, Seoul virus, SEOV, hemorrhagic fever with renal syndrome, viral zoonoses, whole-genome sequencing, pets, rats, viruses, hantavirus, Seoul hantavirus, United Kingdom, Scotland

## Abstract

We describe a case of hemorrhagic fever with renal syndrome caused by Seoul virus in a woman in Scotland, UK. Whole-genome sequencing showed the virus belonged to a lineage characterized by recent international expansion, probably driven by trade in pet rats.

Hantaviruses (genus *Orthohantavirus*, family *Hantaviridae*) are segmented, single-stranded RNA viruses maintained by chronic subclinical infection of rodent and insectivore hosts. Human infection occurs after exposure to the saliva, urine, or feces of infected animals. The infecting viral species determines syndromic manifestation in humans. Hantaviruses endemic to the Americas, such as Sin Nombre virus and Andes virus, cause hantavirus pulmonary syndrome, whereas Old World hantaviruses, mainly Hantaan virus, Dobrava virus, and Seoul virus (SEOV), cause hemorrhagic fever with renal syndrome (HFRS). We identified and treated HFRS in a woman in Scotland, UK.

## The Study

In 2019, a woman 51 years of age sought treatment at Queen Elizabeth University Hospital (Glasgow, Scotland, UK) for fever, diarrhea, vomiting, and malaise that had developed 5 days earlier. A teacher in Glasgow, she lived with her husband and 3 teenage children. Her 12-year-old daughter had experienced a febrile illness associated with myalgia the previous week but recovered without seeking medical attention. There was no notable travel history.

The patient had bred fancy rats (*Rattus norvegicus domestica*) for the previous 2 years. She owned 37 rats, which were housed in cages in her bedroom. Three months before, she had acquired 4 stud rats from a local breeder. She had overseen several recent litters; 27 of her rats were newborn or juvenile. The patient and her 12-year-old daughter cared for the rats, whereas her husband and other children had little contact with the animals.

At admission, the patient had conjunctival suffusion, pallor, and temperature of 38.9°C. We found evidence of mild perioral bleeding. She had a blood pressure of 91/66 mm Hg and a heart rate of 125 bpm.

Blood tests revealed mild lymphopenia, hemoglobin levels within reference levels, and a platelet count of 70 ×10^9^/L (reference range 150–410 × 10^9/L) (Table 1). Her serum creatinine was 87 mmol/L (reference range 40–130 mmol/L). She had transaminitis, but her bilirubin and coagulation results were within reference ranges. She had elevated levels of C-reactive protein (58 mg/L [reference range 1–10 mg/L]). Urine dipstick showed microhematuria and proteinuria. An abdominal ultrasound showed no abnormalities. She had negative serologic results for viral hepatitis and HIV.

**Table 1:** Hematologic and biochemical markers in patient with Seoul virus infection, Scotland, UK, 2019*

Parameter (reference range)	Day 1	Day 2	Day 4	Day 6	Day 8	Day 12	Day 29
Hemoglobin (115–165 × 10^9^/L)	136	144	114	133	112	122	128
Platelets (150–410 × 10^9^/L)	70	38	46	111	180	244	257
Neutrophils (2.0–7.0 × 10^9^ cells/L)	2.3	2.4	2.1	3.6	3.2	3.2	3.5
Lymphocytes (1.1–5.0 × 10^9^ cells/L)	0.7	1.2	1.4	4	2.4	2.5	3
Sodium (133–146 mmol/L)	131	133	143	144	145	142	141
Urea (2.5–7.8 mmol/L)	6.4	6.4	7.4	9.1	9.6	7.4	5.2
Creatinine (40–130 mmol/L)	87	92	158	182	134	97	83
Estimated glomerular filtration rate (>60 mL/min)	>60	56	30	25	36	53	>60
Alanine aminotransferase (<50 U/L)	NA	282	101	108	71	70	17
Aspartate aminotransferase (<40 U/L)	NA	341	73	84	41	35	18
Albumin (35–50 g/L)	NA	35	24	27	26	32	38
C-reactive protein (1–10 mg/L)	58	104	50	30	14	3	NA

*NA, not available.

Our differential diagnosis included leptospirosis, rat-bite fever, and hantavirus infection. We prescribed oral doxycycline and intravenous benzylpenicillin. Her hemodynamic condition stabilized with intravenous fluids during the next 48 hours. However, oliguria and acute kidney injury developed; creatinine levels peaked at 182 mmol/L. Polyuria also developed, and she required intravenous fluid therapy before her renal function began to recover.

Blood and urine cultures yielded no growth. Samples sent to the Rare and Imported Pathogens Laboratory (Porton Down, UK) tested negative for *Leptospira* by enzyme immunoassay and PCR but positive for hantavirus IgG by serologic assay (Table 2). Reverse transcription PCR found hantavirus RNA in patient blood samples. Genomic sequencing matched SEOV small segments in GenBank.

**Table 2:** IgG against hantaviruses in patient with Seoul virus infection, Scotland, United Kingdom, 2019

Virus	IgG titer	
	Day 0 (admission)	Day 29 (convalescent)
Dobrava virus	Negative	>1:10,000
Hantaan virus	>1:10,000	>1:10,000
Puumala virus	Negative	>1:10,000
Saaremaa virus	1:1,000	>1:10,000
Seoul virus	1:3,200	>1:10,000
Sin Nombre virus	Negative	Negative

The patient’s condition improved with supportive therapy, and she was discharged after a 12-day inpatient stay. A public health team inspected her property and recommended that the rats be euthanized, to which she agreed. However, the breeder who supplied the animals removed the rats from the property and refused to cooperate further.

At a clinic appointment 28 days after seeking treatment, the patient had no symptoms. Renal function, liver enzymes, and platelets had normalized, and proteinuria had resolved. Serologic assays demonstrated an increase in IgG titer against Old World hantaviruses (Table 2). We determined the genomic sequences of SEOV small, medium, and large segments by metagenomic sequencing of a stored blood sample from time of admission.

## Conclusions

HFRS is characterized by fever, renal impairment, and thrombocytopenia. HFRS, especially cases associated with Hantaan virus, is responsible for many deaths in Southeast Asia (*1*), whereas HFRS associated with SEOV causes relatively mild disease with a case-fatality rate of <1% (*2*). SEOV cases often begin with fever, malaise, and gastrointestinal symptoms before progressing to shock and acute kidney injury of varying severity (*3*,*4*). Associated transaminitis is suggestive of HFRS caused by SEOV (*2*), as illustrated in this case. Most infections are probably subclinical or mild, as demonstrated by this patient’s daughter, who had experienced mild symptoms suggestive of SEOV infection. However, we were unable to obtain a sample for serologic confirmation.

SEOV is widely distributed because of the ubiquity of its principal host, the *R. norvegicus* rat (*5*). In the United Kingdom, where the virus is established in wild rodents, cases of human disease have been associated with occupational exposure in agricultural workers (*6*,*7*). However, growing evidence exists of SEOV circulation among pet rats. The United Kingdom has a network of pet rat owners who trade rats for breeding. In 2013, SEOV was isolated from pet rats in a breeding colony linked to cases of human infection in the United Kingdom (*4*,*8*). Later, human cases of SEOV associated with ratteries were reported in France (*9*) and the Netherlands (*10*). In 2017, a large SEOV outbreak among pet rat owners was linked to in-home ratteries in the United States and Canada (*11*). We used RAxML (https://github.com/stamatak/standard-RAxML) to conduct a phylogenetic analysis of SEOV sequences derived from rats associated with these outbreaks (*4*,*8*,*9*,*10*,*11*) in addition to the virus sequence from the patient described in this report; these isolates formed a well-supported monophyletic clade (Figure). This clade is distinct from SEOV sequences recovered from wild rodents in Europe and the Americas, suggesting a single introduction and international expansion of this lineage into domesticated rat populations, rather than separate local introductions from wild rats. A recent study from the Netherlands revealed evidence of international trading of rats by breeding farms and private persons, a practice that might promote cross-border dispersal of this lineage (*12*).

**Figure:**
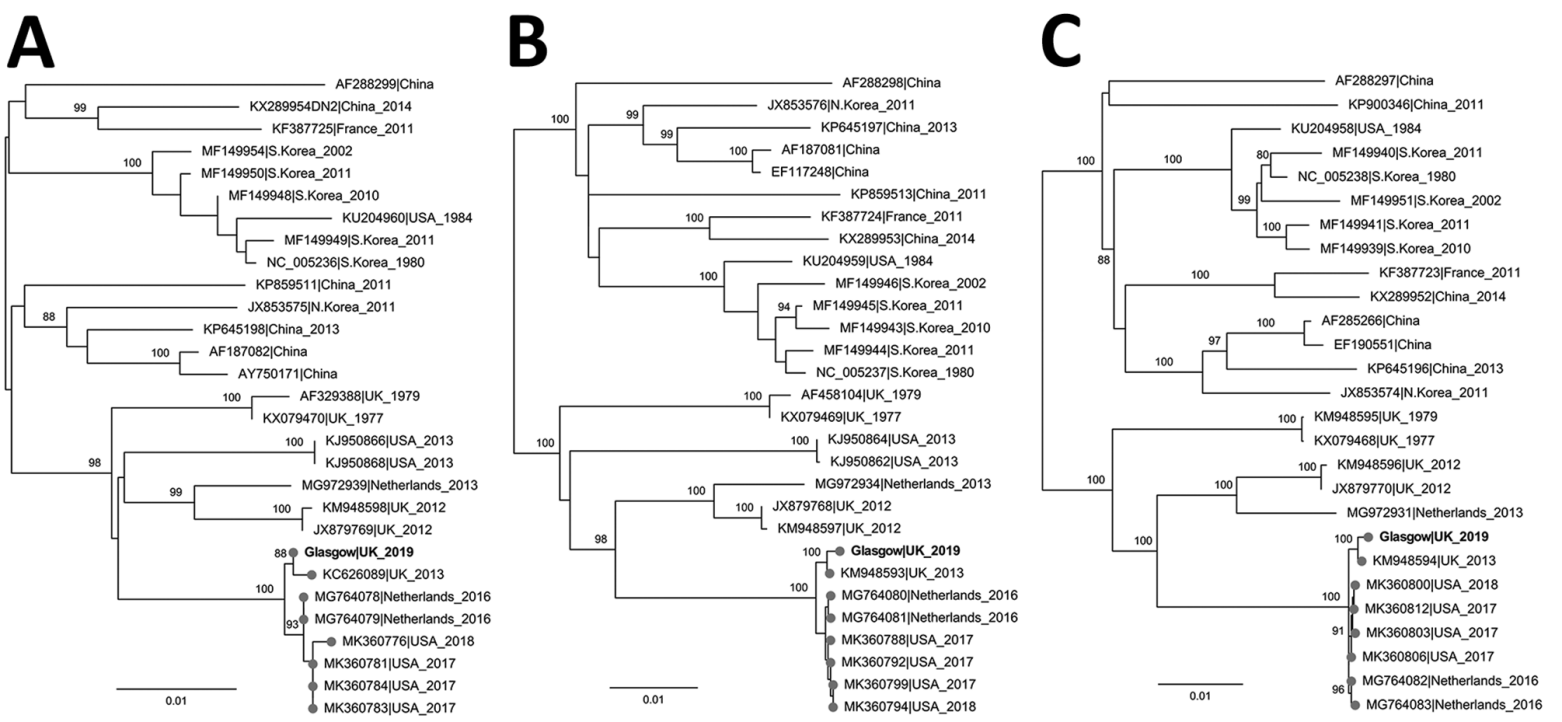
Maximum-likelihood phylogenetic tree based on the small (A), medium (B), and large (C) segments of Seoul virus isolated from a patient in Scotland, United Kingdom, 2020 (bold text; GenBank accession nos. MZ343375–7), and reference sequences. Isolate names indicate GenBank accession number as well as location and date of isolate. Phylogenetic relationships inferred by RAxML (https://github.com/stamatak/standard-RAxML) using the general time-reversible plus gamma distribution plus invariable site model as determined by jModeltest (https://github.com/ddarriba/jmodeltest2). The tree was rooted at midpoint. Numbers to the left of nodes indicate bootstrap values based on 1,000 replicates. Gray circles indicate sequences associated with domesticated rats. Scale bar indicates substitutions per site.

The risk for SEOV transmission from domesticated rats might be greater than that posed by wild rodents. A seroprevalence study performed in 2014 revealed that 34.1% of fancy rat owners in the United Kingdom had antibodies against SEOV, compared with 3.3% of healthy blood donors and 1.7% of farmers (*13*). These findings might reflect the behaviors of owners fancy rats who often view their pets as valued companion animals. In our study, the patient and her daughter reported kissing the rats and housing them in bedrooms. Owners might not follow public health advice on preventive measures such as avoiding kissing or holding small mammals near the face and keeping rodents out of sleeping and eating areas; public health messaging should be tailored to address the specific health beliefs of this community (*14*). Although the patient in this study agreed to the proposed euthanasia of her rats, the breeder preemptively removed them from the property and refused to cooperate further, mirroring the behavior of breeders in other outbreaks (*10*,*15*). This pattern suggests breeders’ rejection of SEOV as a major pathogen (*10*,*15*). A holistic response to future outbreaks might prevent similar situations. For example, an outbreak in the United States was effectively controlled by a mixture of methods including euthanasia, intensive owner education, and test and quarantine approaches that enabled the protection of uninfected rats (*11*).

In summary, we report the persistence of a SEOV lineage associated with pet rats in the United Kingdom, highlighting the ongoing risk for HFRS among pet rat owners. This lineage has undergone recent international dissemination, probably driven by trade of pet rats. Engagement and education within the community of owners and breeders will be crucial to limiting further SEOV infections transmitted from pet rats. Physicians should consider SEOV in any febrile patient with a recent history of rat exposure.
